# Emetics of Ipecacuan. in Hemorrhage

**Published:** 1840-10

**Authors:** 


*Emetics of Ipecacuan. in Hemorrhage.*—Dr. Osborne states that this treatment in menorrhagia has never as yet failed in his hands, except when the progress of the case afterwards proved the formation of scirrhous or cancerous structures of the uterus. "The remarkable effects of emetics of ipecacuanha in restraining hemorrhage," he adds, "is not confined to this organ.. In a case of violent epistaxis, in which several remedies were ineffectual, I tried it while preparations were going on for plugging the posterior nares, and with success, so as to render that measure unnecessary. In hemoptysis, I am unable to add the facts already known respecting its efficacy, being of opinion that hemorrhage from the lungs is always salutary, and that the practice of giving the mineral acids, &c., to discourage it in phthisis is injurious. A very considerable benefit is generally perceptible, after the vessels of the diseased lung have been unloaded by this discharge. When, however, a violent hemorrhage takes place from the lungs, and blood is expectorated in such quantities as to endanger life, then all our efforts must be directed to its suppression. In a late case (not phthisis) I failed in the emetic, but as I lost sight of the patient subsequently, I am unable to pronounce as to the cause of the hemorrhage, and therefore as to the cause of the failure."—*Dublin Journal of Medical Sciences.*—*Boston Med. and Surg. Jour.*

